# A Scrap of History

**Published:** 1873-10

**Authors:** 


### A SCRAP OF HISTORY.

The *Chemist and Druggist*, in a late editorial, acknowledges the indebtedness of science to Paracelsus in these words :—

"This gentleman, who was born in 1493, seemed to carry with him into the sixteenth century a judgment and experience matured by an acquaintance with the practice and theories of the nineteenth. He treated the schools of physic then in existenee with the scorn and ridicule which probably they well deserved, and he went about administering the then almost unknown medicines, antimony, opium and mercury, with such skill and judgment, that his cures and his fame as a physician soon spread all over Europe, without the aid of newspapers, while these medicines, which he may be said to have introduced, have maintained almost exactly the reputation which he assigned to them even unto this day. Such an extraordinary instance of a man living before his time is hardly on record in any branch of learning."

